# Analysis of factors influencing the efficacy of NAC and prognosis between HER2-zero and HER2-low HR negative breast cancer

**DOI:** 10.3389/fcell.2024.1417271

**Published:** 2024-11-22

**Authors:** Jing-Jing Liu, Yi Zhang, Shi-Chao Zhang, Xu Liu, Shu-Nan Wang, Xin-Yu Liu, Jin Zhang

**Affiliations:** ^1^ The Third Department of Breast Cancer, Tianjin Medical University Cancer Institute and Hospital, National Clinical Research Center for Cancer, Tianjin, China; ^2^ Key Laboratory of Cancer Prevention and Therapy, Tianjin Medical University Cancer Institute and Hospital, Tianjin, China; ^3^ Tianjin’s Clinical Research Center for Cancer, Tianjin Medical University Cancer Institute and Hospital, Tianjin, China; ^4^ Key Laboratory of Breast Cancer Prevention and Therapy, Tianjin Medical University, Ministry of Education, Tianjin, China

**Keywords:** HER2-low, NAC, PCR, prognosis, HER2 evolution

## Abstract

**Objective:** The aim of this paper was to assess the differences in clinicopathological characteristics, efficacy and prognosis of neoadjuvant chemotherapy (NAC) in human epidermal growth factor receptor2(HER2)-zero and HER2-low hormone receptor (HR)-negative breast cancer (BC) patients, and the impact of HER2-evolution on prognosis before and after NAC.

**Methods:** 319 triple negative breast cancer (TNBC) patients who completed NAC and surgery from August 2014 to August 2018 at Tianjin Medical University Cancer Institute and Hospital were included. Clinicopathological features, efficacy of NAC and assessment of prognosis were retrospectively analysed. The evolution of HER2-zero to HER2-low after NAC is defined as HER2-gain, the evolution of HER2-low to HER2-zero after NAC is defined as HER2-loss, and HER2 unchanged after NAC is defined as HER2-stable.

**Results:** In HR-negative BC, the pathological complete response (pCR) rate was significantly higher in HER2-zero compared with HER2-low patients, and the difference was statistically significant (38.9% vs 23.2%, *p* = 0.004), but there was no significant difference in the prognosis between the two groups. The overall rate of HER2-evolution after NAC was 19.7%, and there was a significant correlation between HER2-loss and histological grading, whereas HER2-gain was significantly associated with Ki-67 expression. In terms of prognosis, HER2-gain was better compared to the other two groups.

**Conclusion:** In this study, we found that HER2-low HR-negative BC showed different clinicopathological features and response to NAC compared with HER2-zero, as well as HER2-evolution before and after NAC had a significant impact on prognosis.

## 1 Introduction

According to 2020 global data, BC has become the most common malignant tumour worldwide, accounting for 11.7% of all new cancer cases globally, and has also become the leading cause of cancer death in women (Slamon et al., 1989).

HER2 is a proto-oncogene that is overexpressed in 10%–30% of invasive BC (Sung et al., 2021). For more than 20 years, HER2 has been dichotomised into negative and positive (Dent et al., 2007). The concept of HER2-low was proposed by Paolo Tarantino et al., in 2020:HER2-low includes immunohistochemistry (IHC) 1+ and IHC 2+/*In situ* hybridization (ISH) (−) (Tarantino et al., 2020), which was also detailed in the 2022 Chinese Society of Clinical Oncology guidelines for the diagnosis and management of BC (Li and Jiang, 2022), and in 2023, the ESMO expert statement supported the consensus on the definition, diagnosis and management of HER2-low BC (Mathey-Andrews, 2024; Tarantino et al., 2023). Previous clinical trials have found that the addition of trastuzumab and pertuzumab Carsten et al. to adjuvant chemotherapy does not result in a prognostic benefit for patients with HER2-low BC (Fehrenbacher et al., 2020; Schneeweiss et al., 2018). The recently developed aIHCntibody-drug conjugate (ADC) drug, T-DXd, has been approved for the treatment of patients with metastatic HER2-positive BC (Nakada et al., 2019) and it effectively targets HER2-low tumour cells and delivers its potent cytotoxic payload to neighbouring tumour cells via a bystander effect (Ogitani et al., 2016a; Ogitani et al., 2016b). And the results of this trial, DESTINY-Breast04 (NCT03734029), not only revealed the effect of T-DXd on HER2-low BC, but also broke the HER2 dichotomy method (Modi et al., 2022).

The proportion of HER2-low in triple negative breast cancer (TNBC) patients is significant, and the effect of changes in HER2 expression before and after NAC on the prognosis of TNBC is not yet known; therefore, the aim of this study was to evaluate the differences in clinicopathological characteristics, efficacy of NAC and prognosis in patients with HER2-zero and HER2-low HR-negative BC, as well as the effect of HER2-evolution on the prognosis of HER2 before and after NAC.

## 2 Materials and methods

### 2.1 Patients

**FIGURE 1:**
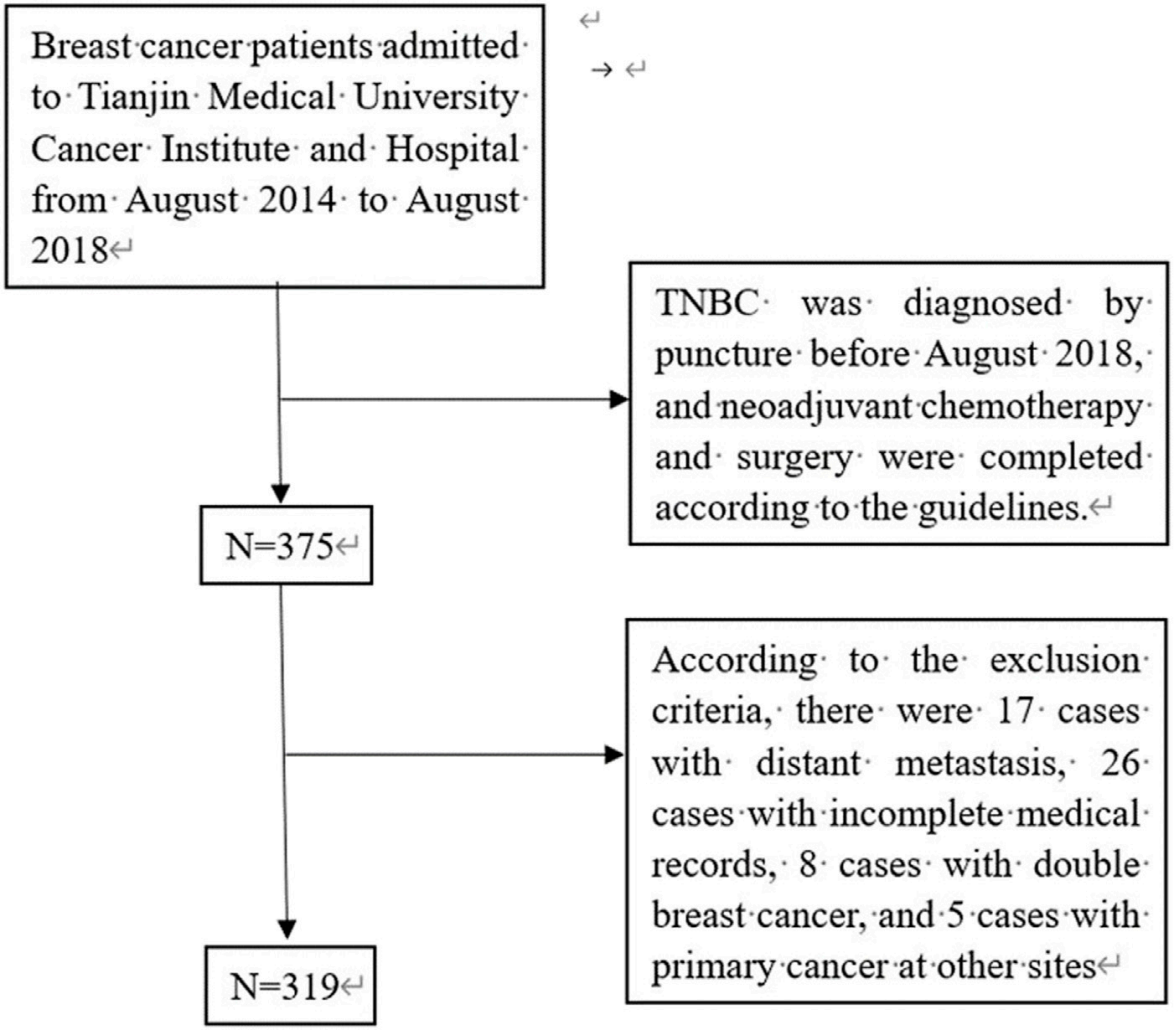
Data screening process.

Patients with pathological tissue confirmed as TNBC obtained by crude needle aspiration biopsy at Tianjin Medical University Cancer Institute and Hospital from August 2014 to August 2018 and completed NAC and surgery according to the guidelines were included, and those with incomplete treatment data or follow-up data were excluded (Figure 1).

Clinical stage was determined by the 8th edition of the American Joint Committee on Cancer (AJCC) tumour-lymph node metastasis (TNM) stage system. Estrogen receptor (ER) and progesterone receptor (PR) were detected by IHC with a cut-off value set at <1%, and HR-negative was defined as negative for both ER and *p*R. HER2 status was determined by the American Society of Clinical Oncology/College of American Pathologists (ASCO/CAP) Guidelines for HER2 testing, and consisted of IHC 0, IHC 1+, or IHC 2+/FISH (−). HER2-zero was defined as IHC 0, and HER2-low was defined as IHC 1+ or IHC 2+/FISH (−). Evolution of HER2-zero to HER2-low by NAC was defined as HER2-gain, evolution of HER2-low to HER2-zero by NAC was defined as HER2-loss, and HER2 unchanged by NAC was defined as HER2-stable.

### 2.2 NAC and efficacy evaluation

All patients in this study were treated with NAC. NAC regimens included anthracycline and paclitaxel-based regimens, paclitaxel combined with platinum-based regimens, and other regimens based on guideline recommendations. During the course of treatment, all patients underwent imaging every two cycles to assess clinical efficacy, including ultrasound and MRI. The vast majority of patients underwent surgery after completing all cycles of NAC. The pathological evaluation criterion pCR was defined (Mazouni et al., 2007) as the absence of invasive carcinoma component in the resected breast lesion and in all lymph nodes ipsilateral to the resected breast after completion of NAC, but residual ductal carcinoma-in-situ component may be present in the breast lesion; or the cancerous cells are necrotic or disappeared, and are replaced by granulation or fibrous tissue (i.e.,ypT0/isypN0).

### 2.3 Statistical analysis

Follow-up by telephone or outpatient review information, with a cut-off date of 31 December 2023, recording the results of the patient’s most recent review, and in case of recurrence/metastasis, recording the time of recurrence/metastasis, i.e., the site of metastasis, etc.,. Disease free survival (DFS) was defined as the time from the end of surgery to the recurrence/metastasis of the disease; Overall survival (OS) was defined as the time from the end of surgery to death or the cut-off of follow-up. Categorical variables described their component ratios, and comparisons between subgroups with different clinicopathological characteristics were performed using the chi-square test. The chi-square test was used for univariate analysis of the relevant factors affecting pCR, followed by multivariate analysis of the relevant factors affecting pCR using logistic regression model, the Kaplan-Meier method was applied to calculate the survival rate and plot the survival curves, and the log-rank test was used to compare the differences in survival between the groups, the above analyses were adjusted for relevant confounding factors such as age, stage, grade, and treatment. And all the data were analysed and processed using SPSS 26.0 statistical software. *p* < 0.05 was considered statistically significant.

## 3 Results

### 3.1 Clinicopathological characteristics

A total of 319 TNBC patients were included in this study. The clinicopathological differences between HER2-zero and HER2-low patients are summarised in Table 1. There were statistically significant differences between the HER2-zero and HER2-low groups in terms of age at diagnosis, clinical N stage, histological grading and Ki67(*p* < 0.05). That is, compared with patients in the HER2-zero group, patients in the HER2-low group had a greater age at diagnosis (*p* = 0.035), a greater proportion of patients with clinical N1 and N2-3 stages at the time of the initial diagnosis (*p* = 0.001), and a significantly higher expression of Ki67(*p* = 0.012), however, the proportion of patients with histological grade III was lower (*p* < 0.001). (Table 1).

**TABLE 1:** Comparison of clinicopathological features between HER2-zero and HER2-low HR-negative BC patients.

Clinicopathological features	n	HER2-zero n (%)	HER2-low n (%)	χ^2^	*p*
Age at diagnosis				4.455	0.035
<50	151	62 (55.4)	89 (43.0)		
≥50	168	50 (44.6)	118 (57.0)		
Menopausal status at diagnosis				0.537	0.464
Premenopausal	162	60 (53.6)	102 (49.3)		
Postmenopausal	157	52 (46.4)	105 (50.7)		
Family history				0.041	0.840
No	292	103 (92.0)	189 (91.3)		
Yes	27	9 (8.0)	18 (8.7)		
Clinical T stage				0.975	0.614
cT0-1	66	23 (20.5)	42 (20.3)		
cT2	158	59 (52.7)	99 (47.8)		
cT3-4	95	30 (26.8)	66 (31.9)		
Clinical N stage				13.194	0.001
cN0	110	53 (47.3)	57 (27.5)		
cN1	167	45 (40.2)	122 (58.9)		
cN2-3	42	14 (12.5)	28 (13.5)		
Pathological pattern				1.523	0.217
Invasive ductal carcinoma	293	103 (92.0)	181 (87.4)		
Other	9	9 (8.0)	26 (12.6)		
Histological grading				55.196	<0.001
Ⅰ-Ⅱ	166	27 (24.1)	139 (67.1)		
Ⅲ	153	85 (75.9)	68 (32.9)		
Lymphovascular Invasion				0.528	0.467
No	252	91 (81.3)	161 (77.8)		
Yes	67	21 (18.7)	46 (22.2)		
TIL (%)				0.239	0.625
<10	278	99 (88.4)	179 (86.5)		
≥10	41	13 (11.6)	28 (13.5)		
Ki67(%)				6.242	0.012
≤14	64	31 (27.7)	33 (15.9)		
>14	255	81 (72.3)	174 (84.1)		
p53 (%)				0.643	0.423
≤10	102	39 (34.8)	63 (30.4)		
>10	217	73 (65.2)	144 (69.6)		
CK5/6				0.215	0.643
Negative	134	49 (43.8)	85 (41.1)		
Positive	185	63 (56.2)	122 (58.9)		
EGFR				0.084	0.772
Negative	88	32 (28.6)	56 (27.1)		
Positive	231	80 (71.4)	151 (72.9)		

### 3.2 HER2 and efficacy of NAC

**FIGURE 2:**
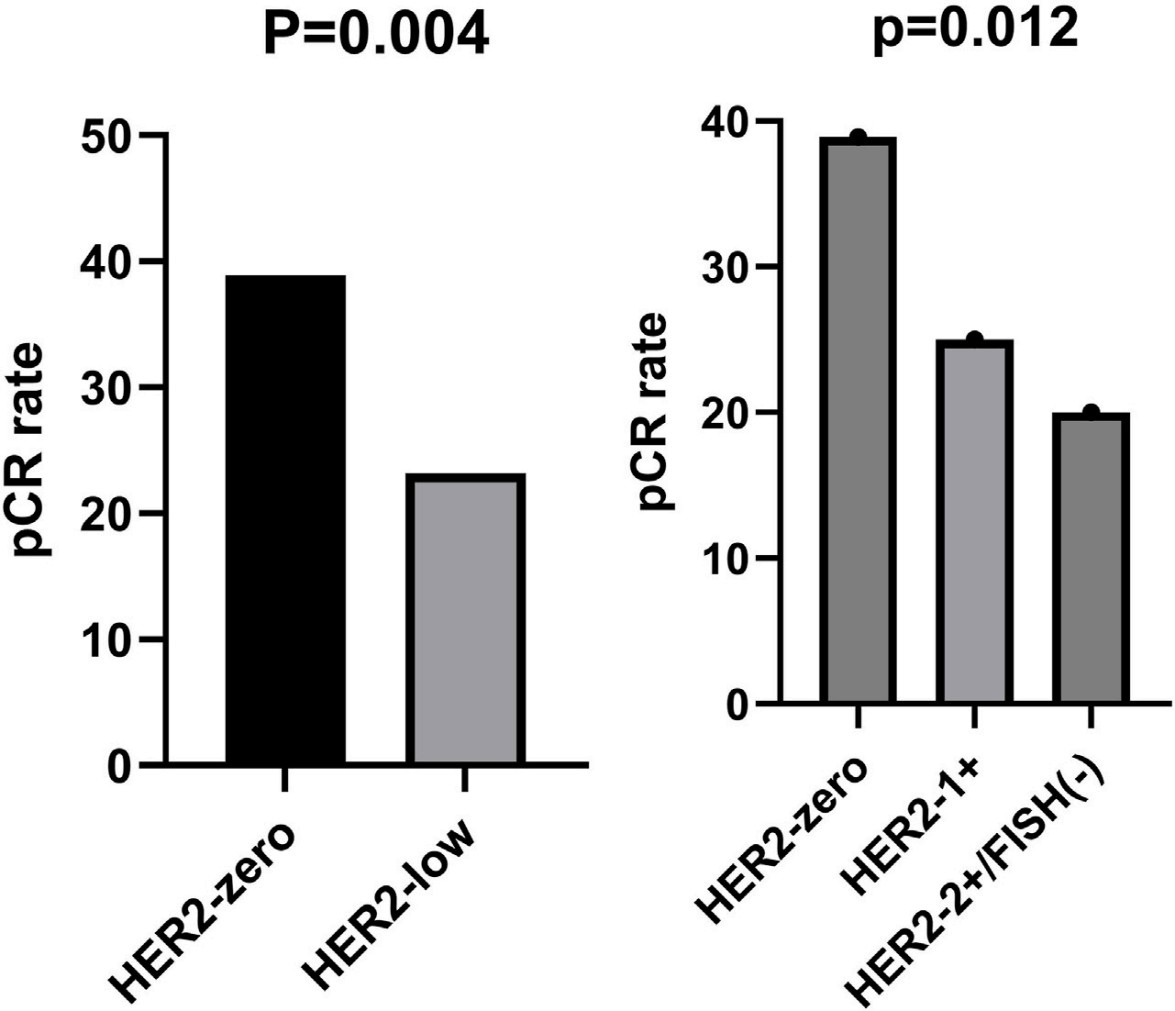
Comparison of pCR rates for different HER2 expression states.

The pCR rate was lower and statistically significant for HER2-low compared to HER2-zero (23.2% vs 38.9%, *p* = 0.004). pCR rates were decreasing between HER2-zero, HER2-1+, and HER2-2+/FISH(−), possessing a statistically significant difference (38.9%vs25% vs 20%,*p* = 0.012). (Figure 2).

In the HER2-low subgroup, the pCR rate was 42.9% in patients with clinical stage T0-1, 22.2% in clinical stage T2, and 12.1% in clinical stage T3-4, with a statistically significant difference (*p* = 0.001). The pCR rate was 25.4% for patients with the pathological type of invasive ductal carcinoma and 7.7% for patients with other pathological types, and the difference was statistically significant (*p* = 0.045). After multifactorial analysis, it was shown that clinical T-stage was an independent influencing factor affecting the pCR rate of NAC in HER2-low HR-negative patients, and the higher the clinical T-stage, the less likely it was to achieve pathological complete remission (Table 2).

**TABLE 2:** Analysis of factors affecting the pCR rate of NAC for HER2-low HR-negative BC.

Factors	n	pCR (%)	χ^2^	*p*	OR (95%CI)	*p*
Age at diagnosis			0.015	0.904		
<50	89	21 (23.6)				
≥50	118	27 (229)				
Menopausal status at diagnosis			0.197	0.657		
Premenopausal	102	25 (24.5)				
Postmenopausal	105	23 (21.9)				
Family history			0.471	0.493		
No	189	45 (23.8)				
Yes	18	3 (16.7)				
Clinical T stage			13.713	0.001		
cT0-1	42	18 (42.9)			1	
cT2	99	22 (22.2)			0.399 (0.182–0.875)	0.022
cT3-4	66	8 (12.1)			0.178 (0.068–0.468)	<0.001
Clinical N stage			5.116	0.077		
cN0	57	15 (26.3)				
cN1	122	31 (25.4)				
cN2-3	28	2 (7.1)				
Pathological pattern			4.009	0.045		
Invasive ductal carcinoma	181	46 (25.4)			1	
Other	26	2 (7.7)			0.231 (0.051–1.037)	0.056
HER2 expression			0.671	0.413		
1+	132	33 (25.0)				
2+/FISH(−)	75	15 (20.0)				
Ki67(%)			0.050	0.823		
≤14	24	6 (25.0)				
>14	183	42 (23.0)				
p53 (%)			0.001	0.979		
≤10	65	15 (23.1)				
>10	142	33 (23.2)				

### 3.3 HER2 and prognosis

**FIGURE 3:**
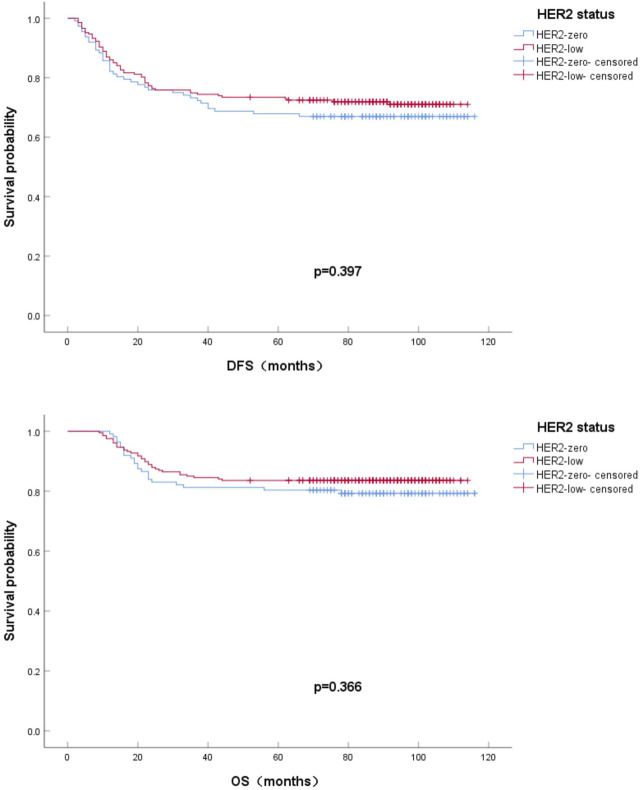
Kaplan-Meier survival curves for DFS **(A)** and OS **(B)** in HER2-zero vs HER2-low HR-negative BC patients.

Among 319 HR-negative/HER2-negative BC patients, 96 cases (30.1%) experienced recurrence or metastasis, of which distant metastasis included, 31 bone metastasis, 16 lung metastasis, 15 liver metastasis, and 8 brain metastasis. The 5-year DFS rates of HER2-zero and HER2-low patients were 67.0% and 72.9%, respectively. By the end of observation, the overall number of deaths was 57 or 17.9%, and the HER2-zero and HER2-low OS rates were 79.5% and 83.6%, respectively. The effects of HER2-low and HER2-zero on survival and prognosis were compared by Kaplan-Meier method of univariate analysis and plotting of survival curves. As seen from the survival curves,the differences in DFS and OS between the two groups were not statistically significant (*p* = 0.397; *p* = 0.366). (Figure 3).

**FIGURE 4:**
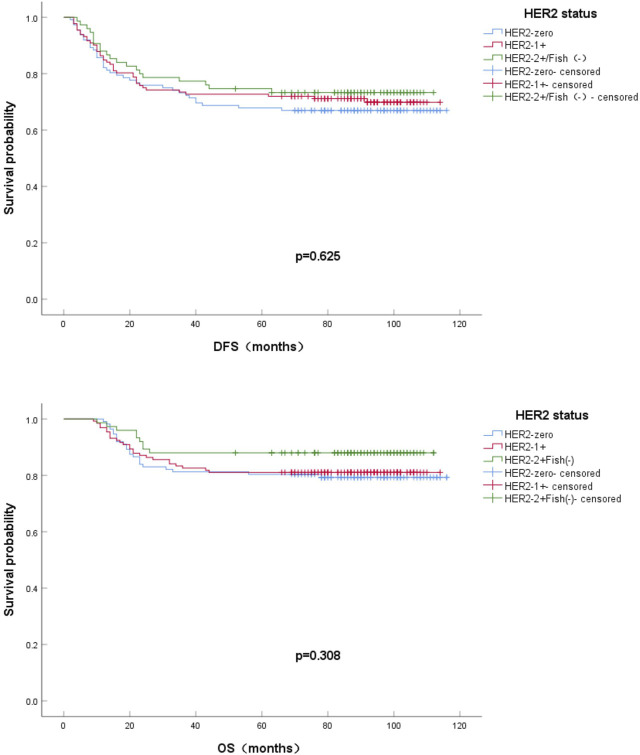
Kaplan-Meier survival curves for DFS **(A)** and OS **(B)** in HER2-zero vs HER2-1+ vs HER2 2+/Fish(-) HR-negative BC patients.

According to HER2 IHC and FISH regrouping, the 5-year DFS rates were 67.0%, 72.7%, and 73.3% for HER2-zero, HER2-1+ and HER2-2+/FISH(−), respectively. By the end of observation, the OS rates were 79.5%,81.1%, and 88.0% for HER2-zero, HER2-1+ and HER2-2+/FISH(−), respectively.The effects of HER2-zero, HER2-1+ and HER2-2+/FISH(−) on survival and prognosis were compared by Kaplan-Meier method of univariate analysis and plotting survival curves. As seen from the survival curves, the differences in DFS and OS between the two groups were not statistically significant (*p* = 0.625; *p* = 0.308). (Figure 4).

### 3.4 Clinicopathological characteristics of HER2-evolution

Of the 319 patients included, the overall rate of HER2-evolution after NAC was 19.7%, with 51 HER2-loss and 12 HER2-gain cases. The relationship between HER2-evolution and clinicopathological features was further analysed, and compared with HER2-stable and HER2-gain cases, a histological classification grade III was higher (43.0% vs. 25.0% vs. 78.4%, *p* < 0.001). Meanwhile, the proportion of Ki-67 > 14% was higher in HER2-gain cases than in HER2-stable and HER2-loss cases (91.7% vs. 82.0% vs. 66.7%, *p* = 0.026). (Table 3).

**TABLE 3:** Clinicopathological features of HER2-evolution in HR-negative BC after NAC.

Clinicopathological features	HER2-stable n = 256	HER2-loss n = 51	HER2-gain n = 12	χ^2^	*p*
Age at diagnosis				1.479	0.477
<50	117	28	6		
≥50	139	23	6		
Menopausal status at diagnosis				0.004	0.998
Premenopausal	130	26	6		
Postmenopausal	126	25	6		
Family history				0.141	0.932
No	135	46	11		
Yes	21	5	1		
Clinical T stage				3.464	0.483
cT0-1	53	9	2		
cT2	131	24	4		
cT3-4	72	18	6		
Clinical N stage				7.297	0.121
cN0	87	21	2		
cN1	131	26	10		
cN2-3	38	4	0		
Pathological pattern				1.777	0.411
Invasive ductal carcinoma	225	48	11		
Other	31	3	1		
Histological grading				24.063	<0.001
Ⅰ-Ⅱ	146	11	9		
Ⅲ	110	40	3		
Lymphovascular Invasion				1.173	0.556
No	203	41	8		
Yes	53	10	4		
TIL (%)				1.696	0.428
<10	220	47	11		
≥10	36	4	1		
Ki67(%)				7.330	0.026
≤14	46	17	1		
>14	210	34	11		
p53 (%)				2.842	0.241
≤10	76	21	3		
>10	180	30	9		
CK5/6				0.575	0.750
Negative	107	23	4		
Positive	149	28	8		
EGFR				0.458	0.796
Negative	69	16	3		
Positive	187	35	9		

### 3.5 HER2-evolution and prognosis

**FIGURE 5:**
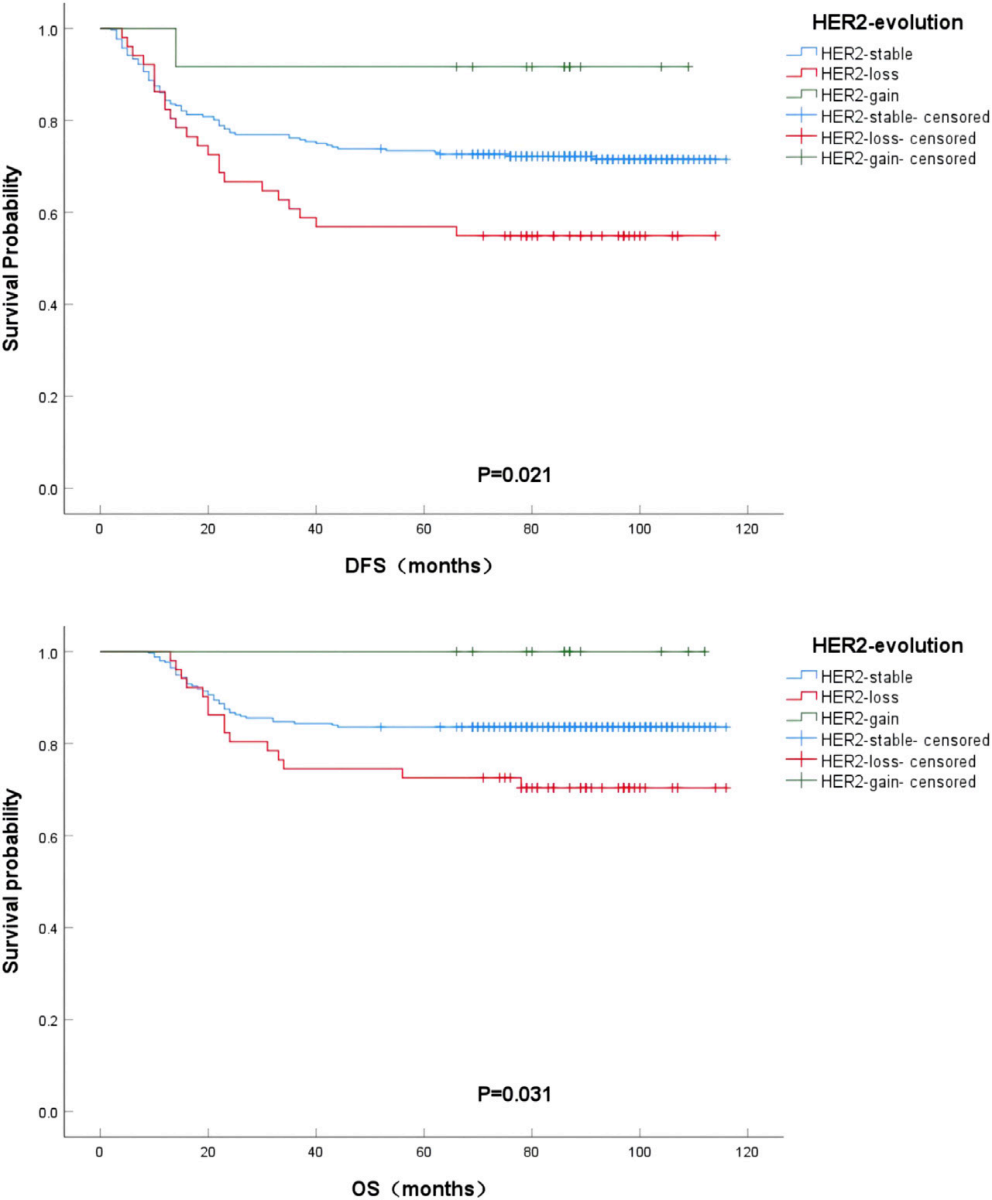
Kaplan-Meier survival curves for DFS **(A)** and OS **(B)** in HER2-stable, HER2-gain, and HER2-loss patients in HR-negative BC.

The prognostic impact of HER2-evolution after HER2-negative BC NAC was compared by Kaplan-Meier method of univariate analysis and plotting of survival curves. From the survival curves, it can be seen that DFS and OS were significantly better in HER2-gain patients compared to HER2-stable patients, but HER2-loss patients had the worst DFS and OS compared to HER2-stable patients, and the difference was statistically significant (*p* = 0.021; *p* = 0.031). (Figure 5).

## 4 Discussion

BC is a heterogeneous disease with significant heterogeneity of cancer cells and tumour microenvironment during its development, so it has different biological features, molecular characteristics and therapeutic features (Zardavas et al., 2015). TNBC is a common type of BC, accounting for about 15%–20% of BC occurrence. This type of BC is a heterogeneous BC lacking expression of ER, PR, and HER2 amplification, and compared with other types of BC that have a higher risk of both local and distant recurrence (Dent et al., 2007; Lejeune et al., 2023). Also due to the lack of relevant receptor markers, patients with TNBC cannot benefit from established endocrine- or HER2-targeted drugs, and therefore the search for new targets and the development of new therapeutic regimens is an urgent issue in this field.

In this study, 64.9% of the patients exhibited HER2-low, and we compared the clinical case characteristics of HER2-low and HER2-zero HR-negative BC patients, which suggested that compared to HER2-zero patients, HER2-low patients were older (*p* = 0.035), had a later clinical N-stage (*p* = 0.001), and had higher Ki67 expression (*p* = 0.012), but histological grading was more (*p* < 0.001). Studies by scholars such as Ximena Baez-Navarro and William Jacot have also validated our results, where among HR-negative patients, HER2-low patients were older and less likely to have grade III histological grading (Jacot et al., 2021; Baez-Navarro et al., 2023). In contrast to our study, some studies have also reported a lower frequency of HER2-low BC Ki67 ≥ 14% or 20% in HR-negative BC compared to HER2-zero tumours (Ma et al., 2024). This may be related to, for example, ethnic differences.

NAC is a frequently chosen treatment option for TNBC and has a high survival rate in patients who achieve pCR (Glück et al., 2012; Liedtke et al., 2023). In our study, we found that among HR-negative BC patients, the pCR rate of HER2-low patients was significantly lower than that of HER2-zero patients (23.2% vs 38.9%, *p* = 0.004), and the results of the unifactorial and multifactorial analyses of the influence of NAC on the pCR rate of HER2-low HR-negative BC suggested that the clinical T-stage was an independent influencing factor. Existing studies are conflicting on this point of view, and although the pCR rates of the two groups are numerically different, fewer are statistically different (Liedtke et al., 2023; Shao et al., 2022). Xu et al. based their study on a multicentre study in China, for HR-negative BC patients, the tpCR [total (both breast and axillary lymph nodes) pathologic complete response] rate, bpCR (breast pathologic complete response) rate, and MP4-5 [The score of Miller-Payne (MP) grade, MP assessment was evaluated based on reduced tumor cellularity of resection samples and comparison with core needle biopsy samples. Grades 4–5 are categorized as good pathological response.] rate of HER2-low patients were significantly lower than those of HER2-zero patients (Xu et al., 2023). Shao et al. similarly explored the effect of the effect of HER2-low on NAC efficacy in different states of HR, and they made adjustments to the definition of pCR, and their results indicated that HER2-low might be related to the pCR of BC patients treated with NAC, but different HR statuses and criteria for the definition of pCR need to be taken into account at the same time (Schettini et al., 2021).

Since HER2-low BC began to receive attention, relevant studies have investigated the differences between HER2-low and HER2-zero. Most of the results showed no significant difference in prognosis between the two when HR expression was not considered (Jacot et al., 2021; Schettini et al., 2021; Horisawa et al., 2022; Agostinetto et al., 2021; de Moura Leite et al., 2021; Gampenrieder et al., 2021; Wu et al., 2023). In the present study, we found that among HR-negative BC patients, the DFS and OS rates were not statistically significant, although HER2-low patients had slightly better DFS and OS rates than those of HER2-zero patients, which was consistent with the results of most of the studies mentioned above; moreover, we did not find any differences in DFS and OS among the HER2-zero, HER2-1+ and HER2-2+/Fish (−) groups. Studies by scholars such as Gampenrieder and William Jacot revealed similar findings in patients with metastatic TNBC and non-metastatic TNBC (Schettini et al., 2021; Gampenrieder et al., 2023). Denkert et al. also reported the prognosis of HER2-low and HER2-zero BC, and according to their study, the prognosis of HER2-low BC was statistically significantly superior to HER2-zero BC, especially in HR-negative patients (Denkert et al., 2021). The difference in outcomes may be related to differences in the number of cases and diagnosis of HER2-low between studies.

In the present study, we found HER2-evolution after receiving NAC, and it has been reported that discordance in HR and/or HER2 status between primary and residual tumours after NAC is a relatively common phenomenon (Miglietta et al., 2022). In this study, we assessed the evolution of HER2 expression from puncture biopsy to residual disease after NAC, and the overall rate of HER2-evolution after NAC was 19.7%, which resulted in a lower figure than in other studies because only BC patients who were HER2-negative both before and after NAC were included in this study. Exploratory analyses of clinicopathological features and HER2-evolution showed that patients with histological grade III were more likely to develop HER2-loss, and those with Ki-67 > 14% were more likely to develop HER2-gain This may be related to the higher tumour heterogeneity in these patients. Unlike the present study, a study exploring the relationship between HER2-evolution and clinicopathological features in TNBC patients with non-pCR after NAC showed that HER2-evolution were more related to clinical T-stage (Shao et al., 2024). Although the results of the two studies were different, they were able to show that HER2-evolution may be associated with higher tumour heterogeneity. To address the impact of HER2-evolution on prognosis, we found that DSF and OS were prolonged in HER2-gain patients compared to HER2-stable, which was more favourable than that of HER2-loss patients. A study by Shao et al. also found that DFS and OS trended more favourably in HER2-gain patients compared to HER2-stable in patients with TNBC, but again, there was no statistically significance (Shao et al., 2024). The impact of HER2-evolution on BC prognosis is unclear in the available studies, and the inclusion and exclusion criteria likewise vary between studies, but focused monitoring of patients with HER2-evolution after NAC is necessary.

Currently, the concept of HER2-ultralow arose as a result of a more nuanced categorization of HER2 expression status, i.e., scoring 0 with incomplete and faint staining in ≤10% of tumor cells (Hayes, 2019). And in Destiny Breast-06, T-DXd was evaluated in patients with HER-low or HER2-ultralow (IHC 0, membrane staining), HR + mBC who had disease progression (PD) after endocrine-based therapy and had not received prior CT for mBC, and it was found that in HER2-low mBC, T-DXd compared to TPC (CT) showed a statistically significant and clinically meaningful PFS benefit and that HER2-ultra-low was consistent with HER2-low results (Franchina et al., 2023). This may suggest a new molecular typing of breast cancer and a new therapeutic strategy for breast cancer patients who were once “drug-free”.

In TNBC patients, although no survival differences in DFS and OS were found, patients with HER 2-low BC had a lower pCR rate than patients with HER2-zero BC. The DESTINY-Breast04 clinical trial demonstrated that in patients with HER 2-low metastatic B, T-DXd resulted in a progression-free survival and OS that was significantly longer than that achieved with the physician’s chosen (Fehrenbacher et al., 2020). Therefore, clinical trials that incorporate HER2-low BC into systemic treatment decisions for non-metastatic breast cancer should be explored.

In conclusion, there is no uniformity in the clinicopathological features and prognosis for patients with HER2-low HR-negative BC. However, as with the results of other studies, the results of this retrospective analysis showed that the clinical case characteristics and efficacy of NAC in HER2-low differ from those in HER2-zero patients, and it is not possible to clarify the clinicopathological and prognostic characteristics of patients with HER2-low HR-negative BC with this study alone. Our study has the advantage of excluding the interference of HR status, but it also has some limitations, including a relatively small sample size and retrospective single-centre study, which may lead to selectivity bias. And different guidelines for HER2 detection and interpretation were used during this period, and lack of pathology focused on correcting HER2 expression. And all the study subjects were Chinese, which should be cautious in interpreting these results because of the possible differences in cancer biology among different races. Our study is also a retrospective study conducted on HER2-low HR-negative BC patients, which we hope will provide a reference for later prospective studies as well as more attention to this population in future clinical work.

## 5 Conclusion

In conclusion, this study found that HER2-low HR-negative BC disorders exhibit different clinicopathological features, response to NAC, and prognosis after treatment, as well as the prognostic impact of HER2-evolution after NAC.

## Data Availability

The original contributions presented in the study are included in the article/supplementary material, further inquiries can be directed to the corresponding author.
